# Genetic alterations in thyroid cancer mediating both resistance to BRAF inhibition and anaplastic transformation

**DOI:** 10.18632/oncotarget.28544

**Published:** 2024-01-24

**Authors:** Mark Lee, Luc GT Morris

**Affiliations:** ^1^Department of Otolaryngology-Head and Neck Surgery, New York Presbyterian Hospital, New York, NY 10032, USA; ^2^Department of Surgery, Memorial Sloan Kettering Cancer Center, New York, NY 10065, USA

**Keywords:** thyroid cancer, drug resistance, anaplastic transformation, BRAF inhibitors, PIK3CA

## Abstract

A subset of thyroid cancers present at advanced stage or with dedifferentiated histology and have limited response to standard therapy. Tumors harboring the *BRAF* V600E mutation may be treated with BRAF inhibitors; however, tumor response is often short lived due to multiple compensatory resistance mechanisms. One mode of resistance is the transition to an alternative cell state, which on rare occasions can correspond to tumor dedifferentiation. DNA sequencing and RNA expression profiling show that thyroid tumors that dedifferentiate after BRAF inhibition are enriched in known genetic alterations that mediate resistance to BRAF blockade, and may also drive tumor dedifferentiation, including mutations in the PI3K/AKT/MTOR (*PIK3CA*, *MTOR*), MAP/ERK (*MET*, *NF2*, *NRAS*, *RASA1*), SWI/SNF chromatin remodeling complex (*ARID2*, *PBRM1*), and JAK/STAT pathways (*JAK1*). Given these findings, recent investigations have evaluated the efficacy of dual-target therapies; however, continued lack of long-term tumor control illustrates the complex and multifactorial nature of these compensatory mechanisms. Transition to an immune-suppressed state is another correlate of BRAF inhibitor resistance and tumor dedifferentiation, suggesting a possible role for concurrent targeted therapy with immunotherapy. Investigations into combined targeted and immunotherapy are ongoing, but early results with checkpoint inhibitors, viral therapies, and CAR T-cells suggest enhanced anti-tumor immune activity with these combinations.

## INTRODUCTION

Thyroid cancer is a disease of generally indolent course with the majority of tumors being of well-differentiated histology and amenable to cure with standard therapy. However, a subset of patients present at more advanced stages or with dedifferentiated histologies that are not cured via standard therapy alone. Dedifferentiated subtypes of thyroid cancer include anaplastic thyroid cancers (ATC) and poorly differentiated thyroid cancers (PDTC), which are thought to arise from a process of gradual microevolution from papillary thyroid cancers (PTC) [1]. Once progressed to the anaplastic form, disease is often incurable with 1-year survival rates as low as 35% with chemoradiation [2, 3]. An improved understanding of the molecular basis of thyroid cancer has led to the development of new targeted agents.

### The MAPK pathway and BRAF inhibition

The mitogen-activated protein kinase (MAPK) pathway regulates processes such as cell proliferation, differentiation and death through a cascade of three sequential protein kinases [4–6]. MAPK pathways are initiated by various extracellular cues such as growth factors or stress resulting in sequential phosphorylation until target substrates in the cytosol and nucleus are activated to alter protein function and gene expression. Mutations involving the cascade can lead to constitutive activation and signal transduction resulting in inappropriate cellular proliferation and tumorigenesis [7].

The mutational landscape of PTC is characterized by several molecular subtypes with mutually exclusive effectors in the MAPK pathway [8, 9]. In PTC, the most common mutations in this pathway are *BRAF* V600E (60%), alterations in *RAS* (15%), *BRAF* non-V600E kinase domain mutations, and mutations of other receptor tyrosine kinases such as *RET*, *NTRK*, and *ALK* (12%). In contrast to PTC, in PDTC, *BRAF* V600E mutations are relatively less common (33% of cases), with alterations in *RAS* and *RET* more common, 28% and 6%, respectively. In ATC, *BRAF* V600E mutations are found in 45% and *RAS* in 24% of cases [10]. In thyroid cancer, the *BRAF* V600E mutation leads to constitutive activation of the RAF/ERK pathway and consequent deactivation of thyroid-specific genes resulting in dedifferentiation and tumor progression [11].

With the limited benefit of conventional chemotherapeutics and radiation and growing evidence of clinical activity of BRAF inhibitors in other cancer types such as melanoma, these agents have been also investigated for efficacy in thyroid cancer. The first agents approved for use in thyroid cancer were sorafenib and lenvatinib. While these agents demonstrated benefits in progression-free survival, they have several limitations including no significant improvements in overall survival [12, 13] and a broad range of toxic effects [14, 15]. Additionally, these agents are multi-kinase inhibitors and not specifically targeted to BRAF [13, 16]. Newer, more targeted agents were subsequently investigated and approved for use in thyroid cancer, including vemurafenib and dabrafenib. These agents are not only targeted to BRAF, but specifically to the V600E mutant oncoprotein [17, 18]. Early trials for these agents were promising: vemurafenib with a median progression-free survival of 18.2 months and 6-month stable disease rate of 35%, [19] and dabrafenib with a median progression-free survival of 11.3 months and partial response and stable disease rates of 29% and 45%, respectively [20]. Additionally, pre-clinical data suggested that BRAF V600E inhibitors may restore radioactive iodine (RAI) uptake in previously refractory tumor cells [18], a finding which bore out in a case series of 10 patients who underwent RAI uptake scan after treatment with dabrafenib, 6 of whom had restored RAI uptake [21]. To date, no phase III clinical trials have evaluated long-term disease control with dabrafenib or vemurafenib monotherapy; however, diverse resistance mechanisms have since been described that explain clinical observations of limited sustained disease control [22].

A number of mutations and compensatory mechanisms have been observed to mediate bypass of BRAF blockade by thyroid carcinoma cells [23]. These mutations may be primary (already present in the tumor) or secondary (acquired over the course of treatment). Examples of these mechanisms include: *PIK3CA* mutations that paradoxically hyperactivate ERK when BRAF is inhibited [24], expansion of subclonal populations harboring *KRAS* mutations leading to constitutive activation of the GTPase and the PI3K/AKT pathway [25], increased autocrine signaling via NRG1 to activate the HER2/HER3 pathway [26], autocrine activation of the c-Met receptor to activate the ERK pathway [27] upregulation of IL-6 secretion with activation of the STAT3/JAK pathway [28], and stimulation of EGFR phosphorylation to reactivate ERK/AKT signaling [29]. Another mechanism of resistance is the transition to an alternative cell state due to selective pressures exerted by targeted therapy [30]. Although rare, this can coincide with dedifferentiation of tumor histology as seen with rare cases of anaplastic transformation after BRAF inhibitor therapy [25, 31].

### Anaplastic transformation in thyroid cancer

**Figure 1:**
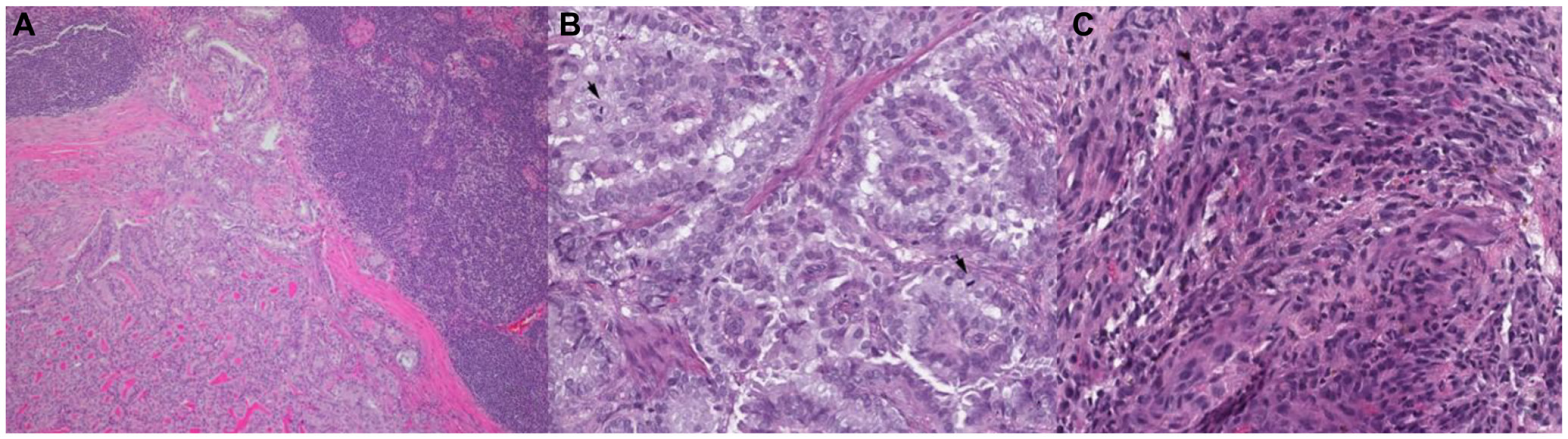
Representative histopathologic slides stained with hematoxylin and eosin of thyroid cancer from well-differentiated to dedifferentiated states. (**A**) 55 year-old-female diagnosed with PTC, specimen taken prior to treatment with targeted therapy, (**B**) 52 year-old-male diagnosed with PDTC, specimen from prior to targeted therapy, (**C**) 66 year-old-male initially diagnosed with PDTC later transformed to ATC on subsequent biopsy (shown here) after BRAF targeted therapy and radiation.

In rare instances, thyroid cancer can evolve from its well-differentiated form, to PDTC—maintaining some semblance of the follicular cells from which it originated—or to ATC, losing all of follicular architecture (Figure 1). Clinical evidence of the microevolution of PTC to ATC began with observations of ATCs occurring in older adults with histories of long-standing or incompletely treated thyroid cancers [32–37]. In addition, the majority—and perhaps nearly all ATCs, accounting for incomplete sampling—have foci of differentiated thyroid cancer, as evidenced by studies utilizing whole-organ sections [38]. Molecular techniques have since supported this conclusion. A study by Wiseman et al. [39] found conserved genomic alterations in adjacent microdissected papillary and anaplastic foci, a finding reflected by data from Hunt et al. [40].

The mechanisms underlying anaplastic transformation are not fully understood however, requiring cytologic and molecular studies for further insight. Ultrastructural analyses have shown that well-differentiated thyroid carcinomas transforming to ATCs undergo loss of tight junctions, desmosomes, and cellular polarity [41]. Molecular alterations found to be associated with ATC and thought to contribute to its pathogenesis include aneuploidy [42, 43], increased copy number alterations [44], and mutations affecting p53 [45–56], bcl-2 [57], cyclin D1 [58], β-catenin [59], Met [60], c-myc [61], Nm23 [62] and Ras [63, 64].

A number of these mutations involve or bypass the MAPK pathway, and together with observations of tumor dedifferentiation after BRAF inhibitor therapy in cancer types such as melanoma [65, 66] and thyroid cancer [25, 67], we hypothesized that mechanisms that drive BRAF inhibitor resistance may overlap with that of anaplastic evolution.

### Mechanisms of BRAF inhibitor resistance and anaplastic transformation may overlap

A recent study from our group [31] observed that the genetic and transcriptomic alterations seen in BRAF inhibitor resistance were also associated with thyroid tumor dedifferentiation. In this study, we assessed mutations in patients with thyroid cancer across the spectrum of tumor dedifferentiation, and genomic and transcriptomic profiles of matched pre- and post-BRAF inhibitor treated thyroid tumors harboring *BRAF* V600E mutations, including a number of cases that dedifferentiated during the course of this study.

**Figure 2:**
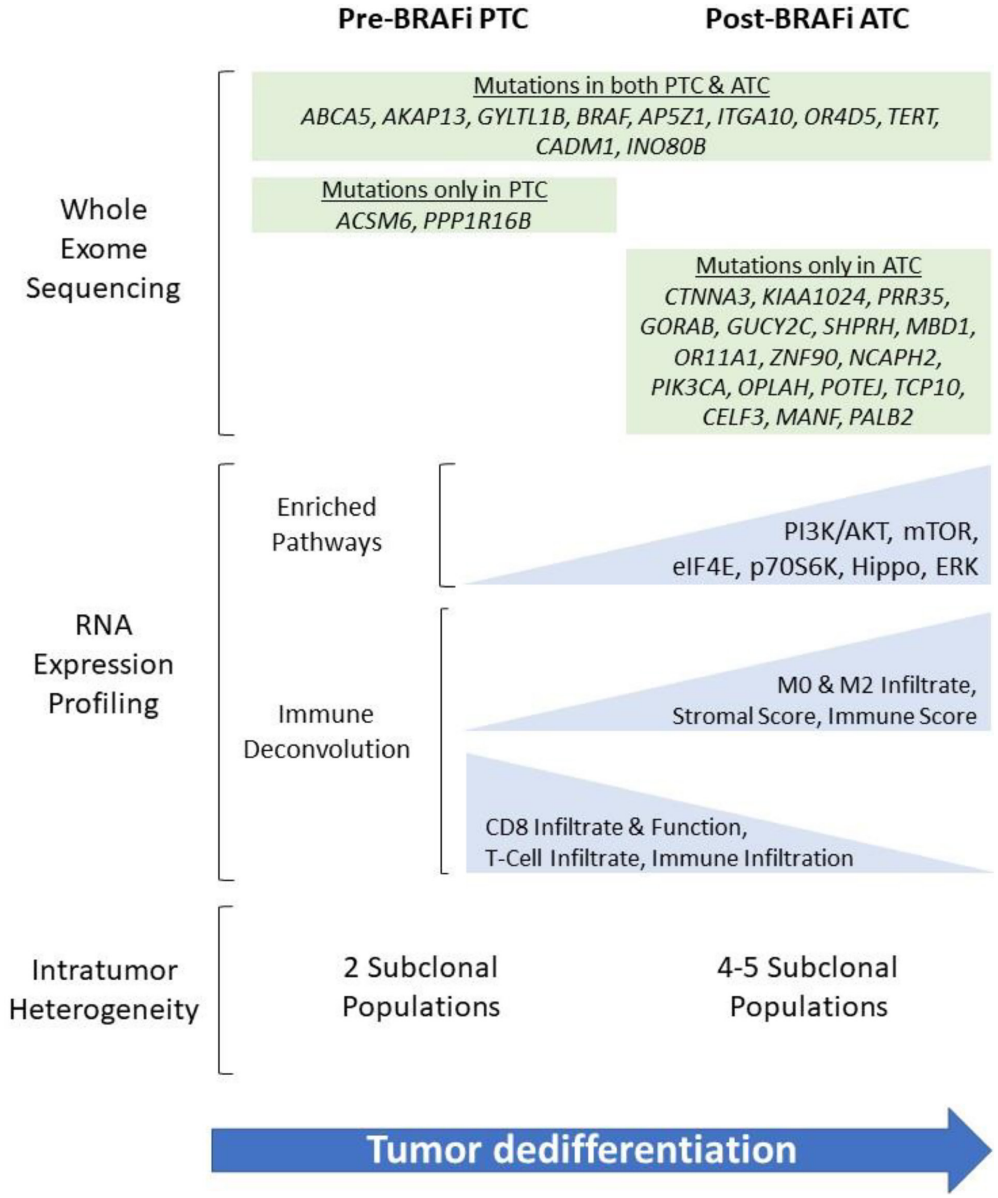
Summary of findings from whole exome sequencing (WES) and RNA expression profiling of tumor samples pre- and post-treatment with vemurafenib and anaplastic transformation in a 55-year-old female initially diagnosed with PTC and subsequently developed biopsy-proven ATC after targeted therapy.

Whole exome sequencing (WES) was performed on matched samples before and after treatment with vemurafenib in a patient initially diagnosed with PTC whose tumor eventually transformed to ATC (Figure 2). Shared mutations were observed between the initial PTC sample and later ATC metastases, consistent with a process of clonal evolution during transformation. Among these shared mutations were the *BRAF* V600E alteration and a *TERT* promotor mutation. Several new mutations emerged in the ATC metastases, notably in 2 genes in the PI3K-AKT-mTOR pathway: *PIK3CA* and *KIAA1024*. Upon further evaluation with orthogonal ultra-deep next-generation sequencing, no mutations in *PIK3CA* were found in any of multiple regions of the initial PTC sample, while the ATC metastases contained the mutation at high cancer cell fractions (>0.90), suggesting that tumor cells harboring the *PIK3CA* mutation may have been present in the initial disease but only in regionally subclonal populations that later seeded the ATC metastases. In fact, we found that the clonal architecture of the ATC metastases was characterized by more subclonal populations than the initial PTC samples.

In this case, because anaplastic transformation occurred after treatment with BRAF inhibition, it is possible that selective pressures exerted by BRAF inhibition contributed to this process.

Mutations in *PIK3CA* have been previously described as a mechanism of resistance to BRAF inhibitors and also associated with the development of anaplastic disease. Using transgenic mouse models of thyroid cancer, Roelli and colleagues [24] showed that double mutants with *Braf* V600E and *Pik3ca* H1047R mutations had paradoxically increased ERK signaling when treated with BRAF inhibitors compared to single mutant mice with *Braf* V600E mutation alone. This phenomenon is likely due to cross inhibition between the PI3K-AKT and MAPK pathways that is essential for appropriate response to proliferative and survival stimuli [68–71]. In turn, the double mutant tumors exhibited decreased response to BRAF inhibitors and more prompt resumption of tumor growth after cessation of therapy compared to single mutant mice. Moreover, the double mutants were noted on histologic examination to have increased size of anaplastic foci compared to untreated mice, suggesting paradoxical ERK signaling via the *Pik3ca* mutation may also drive the development of more aggressive histology. Paradoxical PI3K-dependent ERK activation was also noted in similar experiments using human ATC cell lines [24].

Further supporting the functional relevance of ERK signaling to anaplastic transformation, Ingenuity Pathway Analyses (Figure 2) of the PTC and ATC samples showed enrichment in the PI3K-AKT and mTOR pathways, as well as in an overall measure of thyroid-specific ERK signaling described by Agrawal et al. [9]. Downstream targets such as eIF4E binding protein and p70 ribosomal protein S6 kinase were also enriched. Taken together, these findings support a growing body of literature on the role of compensatory pathways such as PI3K-AKT in bypassing BRAF inhibition, emphasizing the limitations of monotherapy and need for further investigations on multi-modality treatments. These findings suggest that in some cases, selective pressure induced by BRAF inhibition could promote outgrowth of, for example, *PIK3CA*-mutated subclones, and ultimate anaplastic transformation, although it is impossible to say for sure that this would not have happened in the absence of therapeutic BRAF inhibition.

**Figure 3:**
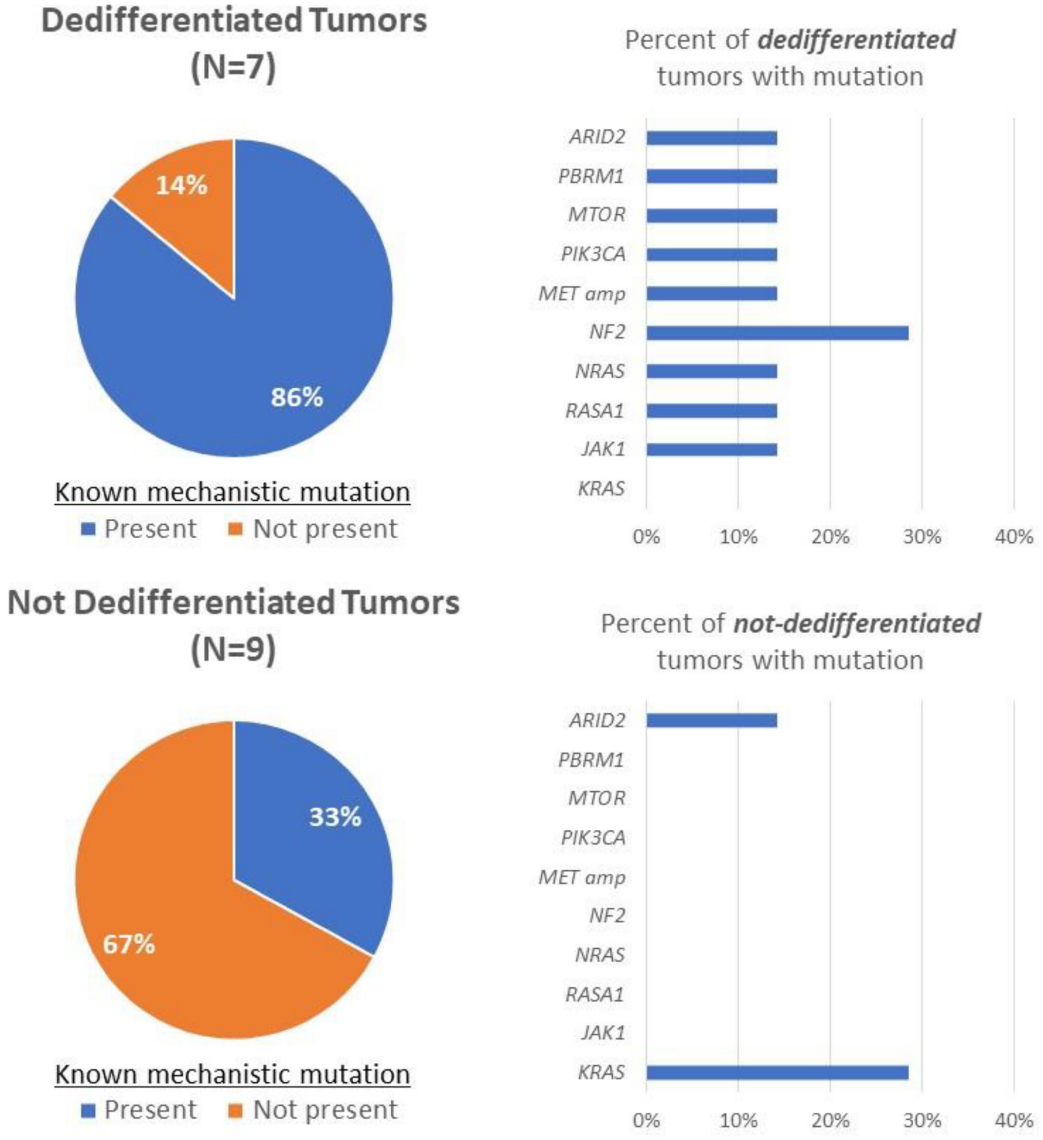
Rates of mutations with previously described mechanistic roles in BRAF inhibitor resistance and/or anaplastic transformation were compared between patients who dedifferentiated after BRAF inhibitor targeted therapy and those who did not using the MSK-IMPACT targeted next-generation sequencing platform.

Other than *PIK3CA*, additional mutations associated with BRAF inhibitor resistance and/or anaplastic transformation have been described in the literature [10, 72–78]. These were also observed to be enriched in thyroid carcinomas that dedifferentiated after treatment with vemurafenib or dabrafenib (Figure 3). Compared to tumors that did not dedifferentiate (*N* = 9), these previously described mutations were found in greater frequency in patients who experienced tumor dedifferentiation (*N* = 7) after treatment (33% vs. 86%). Mutations observed in this cohort include one involved in the PI3K/AKT/mTOR pathway (*MTOR*) as well as additional mutations affecting the MAPK/ERK pathway (*MET* amplifications, *NF2*, *NRAS*, and *RASA1).* Two alternative mechanisms identified in this cohort include the SWI/SNF chromatin remodeling complex (*ARID2* and *PBRM1*) and the JAK/STAT pathway (*JAK1*). The SWI/SNF chromatin remodeling complex has been described to bypass BRAF inhibition by promoting stem-cell-like properties and reestablishing regular thyroid iodine metabolism [10, 75], while *JAK1* mutations mediate resistance to BRAF inhibitors through reduced RN125 expression leading to overexpression of receptor tyrosine kinases such as EGFR [78]. Findings from this expanded cohort highlight the varied number of mechanisms used by tumors to bypass BRAF blockade, again suggesting the need for alternative strategies to address compensatory mechanisms.

### Genetic alterations in 834 thyroid carcinomas along the spectrum of de-differentiation

To assess the role of these mutations in thyroid dedifferentiation more broadly, we examined genetic data from a cohort of 639 advanced, recurrent, and/or metastatic thyroid tumors spanning the spectrum of dedifferentiation that had undergone targeted next-generation sequencing. Here, we provide updated data from an expanded cohort of 834 tumors along the spectrum of thyroid cancer differentiation profiled with targeted next-generation sequencing on the MSK-IMPACT platform (469 PTC, 225 PDTC, and 140 ATC) and compared these to 496 primary PTCs from The Cancer Genome Atlas [9]. The full methodology for MSK-IMPACT was described in a prior article [79]. Briefly, informed consent was collected for analysis of tumor and paired-normal blood samples. DNA was extracted from 834 formalin-fixed, paraffin-embedded thyroid tumors treated at MSKCC between April 2015 and September 2023. Slides were prepared by board-certified subspecialty pathologists with hematoxylin and eosin stains per standard protocol. Histologic type of thyroid cancer was determined according to the World Health Organization’s classification of endocrine tumors [80]. Average target coverage for the PTC, PDTC, and ATC groups were and 530×, 553×, and 646×, respectively. Mutational data for these cohorts are provided in the Supplementary Table 1.

Consistent with prior analyses, the previously identified mutations with described roles in BRAF inhibitor resistance were markedly more prevalent in de-differentiated thyroid carcinoma histologies (Table 1). Mutations in *ARID2*, *JAK1*, *MTOR*, *NF2*, *NRAS*, *PBRM1*, *PIK3CA*, and *TERT* promotor increased to a statistically significant degree in PDTCs and ATCs relative to the TCGA primary PTCs. While not statistically significant, *MET* amplifications are an overall rare event and were only seen in PDTCs and ATCs and not in either PTC cohort.

**Table 1:** Frequency of mutations with described roles in BRAF inhibitor resistance and/or anaplastic evolution across the spectrum of thyroid tumor dedifferentiation

	Primary PTCs^†^	R/M PTCs^‡^	R/M PDTCs^‡^	ATCs^‡^
*ARID2* mutation	1/496 (0.2%)	7/469 (1.5%)^*^	8/225 (3.6%)^***^	9/140 (6.4%)^****^
*JAK1* mutation	1/496 (0.2%)	0/469 (0.0%)	6/225 (2.7%)^**^	2/140 (1.4%)
*MET* amplification	0/496 (0.0%)	0/469 (0.0%)	1/225 (0.4%)	1/140 (0.7%)
*MTOR* mutation	0/496 (0.0%)	4/469 (0.9%)	2/225 (0.9%)	6/140 (4.3%)^****^
*NF2* mutation	1/496 (0.2%)	0/469 (0.0%)	3/225 (1.3%)	19/140 (13.6%)^****^
*NRAS* mutation	34/496 (6.9%)	36/469 (7.7%)	70/225 (31.1%)^****^	23/140 (16.4%)^**^
*PBRM1* mutation	0/496 (0.0%)	1/469 (0.2%)	1/225 (0.4%)	8/140 (5.7%)^****^
*PIK3CA* mutation	2/496 (0.4%)	18/469 (3.8%)^****^	10/225 (4.4%)^***^	20/140 (14.3%)^****^
*RASA1* mutation	1/496 (0.2%)	2/469 (0.4%)	2/225 (0.9%)	1/140 (0.7%)
*TERT* promotor	0/496 (0.0%)	262/469 (55.9%)^****^	126/225 (56.0%)^****^	113/140 (80.7%)^****^

Rates of mutations in primary PTCs compared to recurrent or metastatic (R/M) PTCs, R/M PDTCs, and ATCs. Comparison of rates of mutations by Fisher’s exact test ^*^ *p* < 0.05, ^**^ *p* < 0.01, ^***^ *p* < 0.001, ^****^ *p* < 0.0001. ^†^The Cancer Genome Atlas. ^‡^MSK-IMPACT.

In conclusion, a number of compensatory mechanisms are used by tumors to bypass BRAF blockade. Mutations involving these pathways are noted in higher frequency in tumors that dedifferentiate after BRAF inhibitor treatment. The functional relevance of these pathways is supported by increased expression in alternative pathways such as PI3K-AKT and mTOR after tumor dedifferentiation and BRAF inhibitor treatment.

### The immune microenvironment in anaplastic evolution and BRAF inhibitor resistance

Another area of active investigation is the tumor immune microenvironment because of its known role in thyroid cancer pathogenesis and drug resistance [81–83]. While generally thought to increase anti-tumor immunity [84], BRAF inhibitors may also exert competing effects such as driving tumor infiltration by macrophages, which can mediate resistance to BRAF inhibition through TNFα and VEGF [85]. The development of ATC has also been associated with changes to the immune milieu, including increased infiltration by macrophages [86, 87] and fibroblasts [88, 89], a finding reflected in our prior immune deconvolution analyses comparing ATC samples transformed from PTC after treatment with vemurafenib (Figure 2). In ATC, macrophages form dense nests with elongated cytoplasmic extensions surrounding the undifferentiated tumor cells [87]. The full significance of this interaction is still to be further elucidated. Fibroblasts are enriched in PDTC and are thought to alter the extracellular matrix including enrichment of stromal-derived fibrillar collagen which in turn facilitates mobility of tumor cells [88]. The ATC samples in our study also exhibited increased CD8 T-cell exclusion and dysfunction compared to pre-treatment PTC. Similar changes were observed and further extrapolated on in a recent study by Lu et al. [90] that assessed ATC with single-cell sequencing. The authors described a progression from stress-responsive differentiated tumor cells to a diploid stage with anaplastic cells exhibiting an inflammatory phenotype and lastly to an aneuploid stage with tumor cells gaining mesenchymal properties. This progression parallels a shift from M1 macrophages to M2 and T cells from cytotoxic to an exhausted state. Overall, these changes represent an immune-suppressed microenvironment seen in both BRAF inhibitor resistance and anaplastic evolution, and may represent a potential target for future multi-modality therapy.

### Current practice and future directions in targeted therapy for thyroid cancer

Locally advanced, recurrent, metastatic and/or dedifferentiated tumors occur in a subset of thyroid cancers. The standard therapeutic approach remains surgical resection when feasible, sometimes followed by adjuvant radioactive iodine therapy. There is a limited role for cytotoxic chemotherapy given low response rates, short duration of tumor control, and toxic effects [91]. However, chemotherapy with surgery and radiation can provide marginal survival benefit in stage IVA and IVB ATC [92], and select cases of ATC may be amenable to neoadjuvant use of chemotherapy followed by surgical excision if able [93]. For metastatic disease harboring the *BRAF* V600E mutation, BRAF inhibitors are an available treatment; however, their utility as single-modality therapy is limited by near inevitable disease progression. Recent investigations on BRAF inhibitors have shifted to concurrent blockade of ancillary signaling pathways or promoting anti-tumor immune activity.

Targeted agents have been discovered for several of the resistance mechanisms described above. For patients with ATCs harboring the *BRAF* V600E mutation that are not initially surgically resectable but may be borderline resectable, neoadjuvant combination kinase inhibition with dabrafenib plus trametinib is recommended [94, 95]. This regimen can also be considered for systemic therapy of metastatic ATC based on data from the phase II ROAR trial [96]. The drug everolimus inhibits MTOR and in a limited phase I trial of everolimus with vemurafenib in 20 patients, including 4 with thyroid cancer, it was shown to be well tolerated with partial response and stable disease rates of 22% and 50%, respectively [97]. Crizotinib targets MET and a phase I study including an arm for vemurafenib with crizotinib (*N* = 11) showed a partial response rate of 27% and stable disease of 9%, although none of the groups with clinical benefit included patients with thyroid cancer [98]. PI3K can be targeted with alpelisib or buparlisib, and while no results are available to date for combination use with BRAF inhibitors, they have been tested with the MEK inhibitors trametinib [99] and binimetinib [100] and demonstrated antitumor activity in cancer types such as ovarian cancer. The agent momelotinib (JAK1/2 inhibitor) also has yet to be trialed with BRAF inhibitors, but a study using it in combination with trametinib demonstrated no benefit in *KRAS*-mutated non-small cell lung cancer [101]. Despite the mechanistic rational for multi-target therapies, long-term disease control with these agents remain elusive as demonstrated by pooled analyses with larger samples. For example, the VEM-PLUS study showed no meaningful benefit of vemurafenib with RAF or mTOR-targeted agents compared to vemurafenib alone [102]. Another study using data from 5 phase I and II trials showed no benefit with vemurafenib combined with crizotinib, sorafenib or everolimus [103]. Moreover, these studies found a high rate of adverse effects with combination therapy, requiring dose adjustments that further limit anti-tumor activity. Findings from these recent studies highlight the complex, multifactorial nature of these pathways and the limits of precision oncology platforms.

Based on observations of the immunosuppressive microenvironment of advanced thyroid tumors and prior studies of the possible immune-potentiating effects of BRAF inhibitors, several studies have evaluated the efficacy of combined BRAF blockade with immunotherapy. Indeed, pre-clinical studies of this combination demonstrated possible complimentary activities. A study by Zhi and colleagues [104] using mouse models found that treatment with the BRAF inhibitor PLX4032 in combination with anti-PD-1 antibody reversed TGF-β1/SMAD3 facilitated repression of tumor-specific major histocompatibility complex class II, increased CD4 T-cell infiltration, and suppressed tumor growth. Other novel immunotherapy strategies have also been evaluated for complimentary effects to BRAF inhibitors, including oncolytic HSV [105] and CAR T-cell therapy [106], both of were shown to enhance tumor control in pre-clinical models. However, the pre-clinical literature includes conflicting results, such as a study by Gunda et al. [107] that showed short (2–3 week) tumor control and re-establishment of an immune suppressive environment in ATC mice treated with combination BRAF and immune checkpoint blockade. Investigations into the real-world efficacy of these combinations are ongoing, however early results show promising anti-tumor effects. For example, a phase II clinical trial (ATLEP) on lenvatinib with pembrolizumab including 27 and 8 patients with ATC and PDTC respectively yielded 51.9% partial response and 44.4% stable disease in ATC and 75% partial response and 25% stable disease in PDTC [108]. While promising, additional clinical studies with long-term follow-up are needed to evaluate the real-world efficacy of combined BRAF blockade with these novel immune-modulating therapies.

In conclusion, mutations in thyroid cancer associated with BRAF inhibitor resistance may also be associated with thyroid tumor dedifferentiation. This may be relevant to rare cases of tumors observed to dedifferentiate after BRAF blockade. While early studies of BRAF inhibitors demonstrated anti-tumor activity in advanced thyroid cancers harboring the *BRAF* V600E mutation, subsequent experience and research showed limited long-term disease control due to abundant compensatory mechanisms. One mechanism of resistance is the transition to an alternative cell state driven by selective pressures from BRAF blockade, which on rare occasion can result in tumor dedifferentiation. These mechanisms include mutations affecting the PI3K/AKT (*PIK3CA*, *MTOR*), MAPK/ERK pathway (*MET* amplifications, *NF2*, *NRAS*, *RASA1*), SWI/SNF chromatin remodeling complex (*ARID2*, *PBMR1*) and JAK/STAT pathways (*JAK1*). Dual-target therapies have been trialed but with continued limitations to long-term disease control. Thyroid tumor dedifferentiation and BRAF inhibitor resistance are also found to be associated with a transition to an immunosuppressed state. Early studies on combined targeted and immune-modulated therapy have demonstrated promising outcomes. Further clinical studies are needed to test real-world effectiveness of these novel immunotherapies with targeted therapy.

## SUPPLEMENTARY MATERIALS




