# Supplementary material for: Genetic alterations in thyroid cancer mediating both resistance to BRAF inhibition and anaplastic transformation
Source: Oncotarget. 2024 Jan 24;15:36–48. doi: 10.18632/oncotarget.28544 (PMC10812235; doi:10.18632/oncotarget.28544)
Supplement: Supplementary file 1 [file oncotarget-15-28544-s001.pdf]

Genetic alterations in thyroid cancer mediating both resistance to BRAF inhibition and anaplastic transformation

SUPPLEMENTARY MATERIALS

Supplementary Table 1. Mutational data for 834 thyroid tumors sequenced by MSK-IMPACT. See Supplementary Table 1
